# Atypical Repetition in Daily Conversation on Different Days for Detecting Alzheimer Disease: Evaluation of Phone-Call Data From a Regular Monitoring Service

**DOI:** 10.2196/16790

**Published:** 2020-01-14

**Authors:** Yasunori Yamada, Kaoru Shinkawa, Keita Shimmei

**Affiliations:** 1 IBM Research Tokyo Japan; 2 Poverty and Equity Global Practice The World Bank Washington, DC United States

**Keywords:** dementia, Alzheimer disease, speech analysis, screening, monitoring, behavioral marker, daily conversation

## Abstract

**Background:**

Identifying signs of Alzheimer disease (AD) through longitudinal and passive monitoring techniques has become increasingly important. Previous studies have succeeded in quantifying language dysfunctions and identifying AD from speech data collected during neuropsychological tests. However, whether and how we can quantify language dysfunction in daily conversation remains unexplored.

**Objective:**

The objective of this study was to explore the linguistic features that can be used for differentiating AD patients from daily conversations.

**Methods:**

We analyzed daily conversational data of seniors with and without AD obtained from longitudinal follow-up in a regular monitoring service (from n=15 individuals including 2 AD patients at an average follow-up period of 16.1 months; 1032 conversational data items obtained during phone calls and approximately 221 person-hours). In addition to the standard linguistic features used in previous studies on connected speech data during neuropsychological tests, we extracted novel features related to atypical repetition of words and topics reported by previous observational and descriptive studies as one of the prominent characteristics in everyday conversations of AD patients.

**Results:**

When we compared the discriminative power of AD, we found that atypical repetition in two conversations on different days outperformed other linguistic features used in previous studies on speech data during neuropsychological tests. It was also a better indicator than atypical repetition in single conversations as well as that in two conversations separated by a specific number of conversations.

**Conclusions:**

Our results show how linguistic features related to atypical repetition across days could be used for detecting AD from daily conversations in a passive manner by taking advantage of longitudinal data.

## Introduction

As the world’s elderly population increases, the number of people living with dementia is rising rapidly, making dementia an increasingly serious health and social problem. As of 2018, approximately 50 million people globally were living with dementia, corresponding to about 7.3% of the world’s over 65-year-olds [1]. The total worldwide cost of dementia has risen significantly, being estimated to reach over US $1 trillion in 2018 [1]. At the same time, diagnostic coverage worldwide remains so low that only 40% to 50% of people with dementia have been diagnosed, even in high-income countries [2]. The low-diagnosis coverage makes it more difficult for many patients and their families to receive appropriate support and care. Taking this into consideration, health monitoring technology and services are expected to help increase the diagnosis coverage by detecting signs of cognitive decline resulting from dementia in everyday situations [3,4].

One of the clues for detecting cognitive decline resulting from Alzheimer disease (AD) in everyday situations can be obtained by identifying the evolution of a patient’s language as their AD progresses. While the most typical symptom of dementia is memory impairment due to the medial temporal lobe shrinking [5,6], both retrospective analysis and prospective cohort studies have shown that language dysfunctions prevail even from the presymptomatic period [7,8]. Moreover, studies on pathologically proven AD patients showed that they exhibited syntactic simplification and impairment in lexical-semantic processing [9,10]. A growing body of studies on probable AD patients has also shown that many aspects of speech and language, including grammatical and informational content as well as acoustic characteristics such as the pitch contour, show deficits as AD progresses [4].

Previous computational studies attempted to measure these language dysfunctions in AD patients on the basis of such findings by using acoustic, prosodic, and linguistic features [10-34]. For example, the short-term memory loss attributed to dementia often makes normal conversation difficult due to language dysfunctions such as difficulties with word-finding and word-retrieving [35,36]. These language dysfunctions have been measured by tallying pronoun frequency and fillers, including nonwords and short phrases (eg, “umm” or “uh”) [13,37,38]. The reduction in speech expressiveness is another language dysfunction typically observed in AD patients. This reduction is measured by the decrease in adjectives and indicators related to vocabulary richness [11,39]. Using a combination of these features, previous studies have succeeded in differentiating healthy controls and AD patients [22,37]. However, they mainly investigated speech data obtained while participants took part in neuropsychological tests such as verbal fluency, story recall, and picture description tasks [40]. Whether and how we can measure language dysfunctions resulting from AD during daily conversations remains largely uninvestigated. Because the conversational content and cognitive workload vary in daily conversations where people do not engage in specific tasks, we need to reinvestigate what kinds of speech features could be useful for identifying AD patients. In terms of the language dysfunctions of dementia patients in everyday conversations, previous observational and descriptive studies reported atypical repetition of words and topics as a prominent characteristic [41]. While this repetition has been typically reported to occur in the same conversation, it also appears in separate conversations that may be held on different days. Because it has not been objectively measured, investigating how word and topic repetition differs in AD patients is a promising first step.

In this study, we analyzed the conversational data of seniors with and without AD obtained from longitudinal follow-up in a regular monitoring service. We used natural language processing techniques to automatically measure atypical word and topic repetitions and then investigated whether and how characteristics in topic repetition differed between seniors with and without AD. The results indicated that both linguistic features related to word and topic repetition in paired conversations on different days had better discriminative powers compared with other linguistic features used in previous studies on connected speech data during neuropsychological tests. We also found that they peaked at a specific interval day (around seven days), not at a specific number of conversations. On the basis of these results, we demonstrate how quantifying atypical repetition in continuous and passive monitoring of daily conversations can help automatically detect AD.

## Methods

### Data and Participants

**Figure 1:**
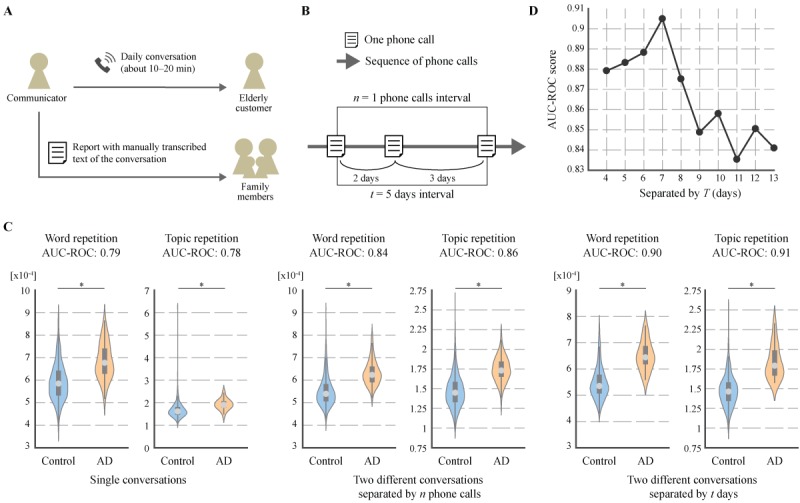
Atypical repetition in conversational data from regular monitoring service. A. Overview of regular monitoring service. The manually transcribed text of the conversation was analyzed in this study. B. Schematic illustrating the paired samples of conversations separated by t days and n phone calls to extract repetition features across different conversations. C. Repetition features of seniors with and without AD. Violin plot is used to visualize the distribution of the data and its probability density. On each side of the violin is a kernel density estimation to show the distribution shape of the data. The wider portion of the violin indicates the higher density and the narrow region represents relatively lower density. The grey box with the whiskers in the violin is the boxplot. The box denotes the 25th (Q1) and 75th (Q3) percentiles. The whiskers denote the upper and lower adjacent values that are the most extreme within Q3+1.5(Q3-Q1) and Q1-1.5(Q3-Q1), respectively. The white dot in the box represents median value. Significant differences are denoted with asterisks (*P<.001). D. AUC-ROC score of topic repetition feature with different T days.

We used conversational data obtained during phone calls with a regular monitoring service for seniors provided by Cocolomi Co, Ltd (Figure 1A). In this service, communicators call and talk with older individuals once or twice a week, typically for 10 to 20 minutes of free conversation. All communicators received training and communicated with participants in accordance with the company guidelines for encouraging participants to speak. In this service, one communicator is typically assigned to a participant, and that participant talks with the same communicator. They manually transcribe the conversations in a spoken-word format by omitting incomplete words and fillers and forward the texts to family members such as their children. They used either home phones or smartphones. All conversations were conducted in Japanese. We analyzed these transcribed text data. All participants agreed to having their conversational data used for research purposes. This study was conducted under the approval of the ethical committee of Shizuoka University and performed in accordance with the Ethical Guidelines for Medical and Health Research Involving Human Subjects.

The data were obtained from 15 Japanese people (12 females and 3 males aged 61 to 91 years; mean 76.8 [SD 9.4] years). Of these, 2 females had received the diagnosis of AD. The morbidity information for other diseases was not available. The communicators knew the diagnosis status of the participants, but they communicated with them in accordance with the company guidelines and did not change conversation methods on the basis of the diagnosis status. The follow-up period ranged from 1 to 33 months (mean period 16.1 months). We analyzed data from 1032 phone calls in total, and the number of phone calls for each participant ranged from 4 to 226 (mean 68.8 phone calls). The average duration of a single phone call for each participant ranged from 6.8 to 22.2 minutes (mean time between participants 12.1 [SD 4.1] minutes). The total call time of our dataset was around 221 hours. Transcribed text data consists of only text spoken by participants, not communicators. The text data contained 1098 characters on average, and we analyzed 1,132,935 characters in total. Table 1 provides the overview of our dataset.

**Table 1:** Demographics of conversational data participants.

Status and gender		Age (years)	Duration (months)	Calls, n	Call time (min), mean (SD)	Number of characters, mean (SD)
**Control**						
	F	61-62	11	31	7.8 (2.0)	422.7 (121.4)
	F	63-64	14	32	14.0 (5.1)	1078.3 (369.8)
	F	75-77	25	75	11.3 (8.5)	744.8 (215.5)
	F	75-75	1	4	12.8 (3.1)	1365.0 (130.6)
	F	78-78	9	64	10.3 (2.0)	815.2 (173.8)
	F	79-80	6	23	11.7 (3.2)	1507.5 (457.4)
	F	80-83	33	109	16.6 (4.7)	1499.7 (391.7)
	F	82-83	19	72	6.8 (3.0)	811.2 (367.9)
	F	88-89	17	104	11.2 (4.4)	944.5 (314.2)
	F	91-91	9	35	22.2 (3.7)	2405.8 (433.2)
	M	63-65	16	72	11.9 (3.1)	1146.8 (242.9)
	M	67-70	33	132	10.6 (2.3)	999.4 (236.5)
	M	82-85	30	226	17.7 (6.3)	1207.0 (497.7)
**Alzheimer disease**						
	F	83-84	14	40	9.5 (2.8)	923.9 (409.2)
	F	85-85	5	13	7.8 (1.8)	662.0 (199.1)
Mean (SD)		76.8 (9.4)	16.1 (10.2)	68.8 (57.2)	12.1 (4.1)	1098.0 (500.3)
Total		—	—	1032	13,230 (221 hours)	1,132,935

### Extraction of Linguistic Features

From each item of phone call data, we extracted linguistic features related to word and topic repetition within single conversations, as well as the standard linguistic features used in previous studies to build models for AD screening from connected speech data during neuropsychological tests (for the list of features, please see the Standard Linguistic Features section) [10,14-34]. In addition, we extracted features related to word and topic repetition across different conversations from the text data of two phone calls separated by *t* days and *n* phone calls interval, respectively (Figure 1B). Specifically, we used all possible pairs of two phone call data items separated by *t* days (*T*–*M*<*t*≤*T*+*M*) for specific days interval and *n* phone calls (*N*–*M*<*n*≤*N*+*M*) for specific phone calls interval. Optimal parameters for *T* (4,5,…,13) and *N* (3,4,…,12) were selected by performing a grid search for each feature. In this study, we used *M*=3, *T*=10, and *N*=7 for word repetition, and *T*=7 and *N*=9 for topic repetition.

For preprocessing, we used the Japanese morphological analyzer MeCab [42] to perform word segmentation, part-of-speech tagging, and word lemmatization on the transcribed texts. Words tagged as numerals or symbols were excluded from the analysis. Predefined stop words were also eliminated.

### Word and Topic Repetition Features

We measured word repetition by using the feature focusing on the number of distinct words being used in a target document. Specifically, the feature was calculated by the inverse number of Honoré’s statistic (HS) [43], defined as HS=100log*V*/(1–*V*_uni_/*U*), where *V* is the total number of words, *U* is the total number of distinct word types, and *V*_uni_ is the number of total distinct word types used only once. For the feature related to word repetition across two text data of different conversations, we combined the data of both texts and then extracted the feature from it.

For the features related to topic repetition, we used a biterm topic model (BTM) [44]. While conventional topic models such as latent Dirichlet allocation have typically been used for estimating the latent topics of conversations [45], BTM was designed to extract latent topics from a limited amount of context, such as that found in tweets and news headlines [44]. Accordingly, we used BTM for estimating the topic in sentences and then extracted features related to topic repetition within conversations. Specifically, we first divided a text document into sentences by using punctuation marks. We then applied a BTM to a set of all the sentences, extracted word probability vectors for each *K* topic and obtained topic proportions allocated to each *l* sentence. For example, when a text document consisted of *L* sentences, we obtained *L*–*l*+1 vectors for topic proportions. We calculated pair-wise similarities with Euclidean distance and used the inverse number of the mean value as topic similarity features within single conversations. For the topic similarity features across two different items of text data *D_i_* and *D_j_*, we calculated pair-wise similarities between topic proportions in *D_i_* and *D_j_* and used the inverse number of the mean value. The parameters *K*=200 and *l*=3 were used.

We briefly describe how BTM works. In contrast to conventional topic models such as latent Dirichlet allocation [45], BTM directly models the generation of word co-occurrence patterns in the whole corpus to estimate topics from a small amount of context in a document [44]. The key concept is that if two words co-occur more frequently, they are more likely to belong to the same topic. The term *biterm* denotes an unordered word pair occurring in a short text. For example, a document with three distinct words generates three biterms:

(*w*_1_, *w*_2_, *w*_3_)⟹{(*w*_1_, *w*_2_), (*w*_2_, *w*_3_), (*w*_1_, *w*_3_)}.

In general, a document containing *n* distinct words generates *n*C_2_ biterms. The biterms are typically extracted from each sentence, and by combining them we get a corpus *B* for the target document as a set consisting of ‖*B*‖ biterms. With the BTM, it is assumed that each topic is a multinomial distribution over the biterm (*ϕ*), and the whole corpus is a mixture of these topics (*θ*). The process of extracting biterms can be conducted as follows:

1. Draw a topic distribution θ ~ Dirichlet (α) for the whole corpus.
2. Draw a topic-specific word distribution ϕk Dirichlet (β) for each topic k.
3. For each biterm bi in the whole biterm set: Draw a topic assignment z ~ multinomial (θ). Draw two words wi,1, wi,2 ~ multinomial (ϕZi).

**Figure 2:**
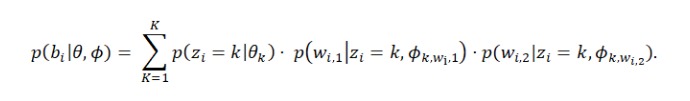
The equation of the probability of biterm bi.

Following the above procedure, the probability of biterm *b_i_*=(*w_i_*,_1_, *w_i_*,_2_) conditioned on the model parameters *θ* and *α* can be written as seen in Figure 2. For more details, we refer the reader to the original paper [44].

### Standard Linguistic Features

On the basis of related work on connected speech data during neuropsychological tests, we extracted 29 linguistic features categorized into 4 types of linguistic features that would be useful for inferring AD: parts of speech, vocabulary richness, syntactic complexity, and perseveration [10,14-34]. Here, neuropsychological tests in previous studies include a picture description task using a picture of the Cookie Theft, comprehensive aphasia test, and the Wechsler Logical Memory tests [46-48].

The first feature set, related to parts of speech, consists of 14 features: frequency and the ratio of part-of-speech tags. We extracted the part-of-speech information by using Mecab [42] and then computed the frequency of occurrence of different parts of speech (nouns, verbs, adjectives, auxiliary verbs, and conjunctions). We also computed ratios, namely, each part of speech normalized by the total number of word tokens in the document. Analyzed part-of-speech tags included nouns, verbs, adjectives, pronouns, adverbs, auxiliary verbs, and conjunctions. We also calculated the ratio of noun to verb and the ratio of pronoun to noun.

The second feature set, related to vocabulary richness, consists of 3 features: type-token ratio (TTR), Brunét’s index (BI), and HS [11,43]. This feature set measures lexical diversity, which tends to decrease in AD cases [11,13]. TTR compares the total distinct word types (*U*) to the total word count (*V*) as TTR=*U*/*V*. Using the same *U* and *V*, BI is defined as BI=*V^U^*^–0.165^. Unlike other measures related to vocabulary richness, for this measure, the lexical richness becomes greater as BI becomes smaller. HS gives particular importance to unique vocabulary items used only once, also known as hapax legomena *V_uni_*. HS is defined as HS=100log*V*/(1–*V_uni_*/*U*).

The third feature set, related to syntactic complexity, consists of 7 features related to length metrics as well as dependency relations. For features related to length metrics, we calculated 4 measures consisting of mean length of sentences, total number of sentences in a document, total number of words in a document, and total number of characters in a document. For features related to dependency relations, we calculated total number of dependencies in a document, the average dependencies per sentence, and total dependency distance in a document. As for the dependency structures, we used CaboCha [49]. Dependency distance was calculated by the sum of all the linear distances between 2 syntactically related words in a sentence. This dependency distance has been considered an important index of memory burden and an indicator of syntactic difficulty [50].

The final feature set, related to perseveration, consists of 5 features. First, sentences were converted into term frequency–inverse document frequency (TF-IDF) vectors by using a bag-of-words model [51]. TF-IDF is a numerical statistic intended to reflect how important a word is to a document. By using TF-IDF vectors, we then calculated the cosine similarity between sentences [51]. We then calculated the proportion of sentence pairs equal to 0 and below 2 thresholds (0.3, 0.5) as well as the minimum and average cosine distances across all pairs of sentences.

### Statistical Analysis

All statistical analyses were done in the R environment (R Foundation for Statistical Computing). A 2-tailed Student *t* test was applied for each feature to determine significant differences of enrichment between the two populations, and the resulting *P* values were adjusted with Bonferroni multiple testing correction. Adjusted *P* values below .01 were considered significantly different.

## Results

We first investigated whether and how each of the 6 types of features for measuring repetition differed between seniors with and without AD: 2 repetition types (word and topic) × 3 data sources (single conversations, 2 different conversations separated by *t* days, and 2 different conversations separated by *n* phone calls). The discriminative power was measured by using both effect size (Cohen *d*) [52] and AUC-ROC. For Cohen *d*, an effect size of 0.80 is considered large, 0.50 is medium, and 0.20 is small [52]. ROC is a graphical plot that illustrates the diagnostic ability of a binary classifier system that ranges from 0 to 1.

Results showed that all 6 features had significant increased repetition in the AD group with large effect size (*P*<.001, 2-sided *t* test with Bonferroni multiple testing correction; Cohen *d* >0.80; Figure 1C). When we compared the discriminative power among them, the feature related to topic repetition across 2 conversations on different days showed the highest AUC scores and largest effect size (AUC=0.91); effect size of –1.76, 95% CI –2.15 to –1.36; Table 2). In addition, the features for word and topic repetition across different conversations separated by specific days interval and phone calls interval had larger effect size and higher AUC scores compared with those in single conversations (Table 2). We also found that the features extracted from conversational data at *t* days interval had better discriminative power than those at *n* phone calls interval (Table 2).

To deepen our understanding of the nature of repetition features across different days, we investigated the relationship between the discriminative power of the repetition features and interval days of paired conversations. Results showed that after they increased in the beginning, they peaked at around *T*=7 days and then had a tendency to decline (Figure 1D). This suggests that the difference of topic repetition between seniors with and without AD might change with a certain tendency, rather than randomly, with intervening days for two different conversations, and be the largest in the medium interval.

We next compared the repetition features with the standard linguistic features that were typically used in previous studies on connected speech data during neuropsychological tests [10,14-34]. Specifically, we extracted a total of 29 features used in previous studies: 14 features related to part-of-speech distribution, 3 features related to vocabulary richness, 7 features related to syntactic complexity, and 5 features related to perseveration. We found that 13 of the 29 features showed a significant difference between healthy control and AD groups in our dataset of daily conversations (*P*<.01, 2-sided *t* test with Bonferroni multiple testing correction; 11 out of 14 features relating to part-of-speech distribution, 1 out of 3 features relating to vocabulary richness, 1 out of 7 features relating to syntactic complexity, 0 out of 5 features related to perseveration; for exact *P* values see Multimedia Appendix 1). Through a comparison of discriminative power, we found that both topic and word repetition features across two conversations on different days outperformed all 29 features used in previous studies in terms of AUC-ROC scores (Table 2 and Multimedia Appendix 1), followed by conjunction ratio (AUC=0.89; effect size of –2.03, 95% CI –2.33 to –1.73) and pronoun to noun ratio (AUC=0.87; effect size of –1.94, 95% CI –2.24 to –1.64). These results indicate that topic and word repetition across daily conversations on different days might be a better indicator for differentiating AD than linguistic features used for inferring AD in neuropsychological test settings.

Finally, we investigated how changes in the language function of AD patients measured by linguistic features are different during neuropsychological tests and daily conversations. First, we summarized the linguistic features reported to be significant in distinguishing AD from healthy controls in previous studies on connected speech data during neuropsychological tests [10,14-34]. For the selection of the previous studies, we referred to the review article [40] as the baseline and added studies. Specifically, we considered only studies based on a group comparison with a group of healthy controls. Only studies focusing on connected speech through neuropsychological tests including picture descriptions have been considered. Semistructured or unstructured interviews including open-ended questions were not included. The features reported as significant in at least half of the studies were considered as the significant features. We then compared them with the results of statistical analysis on our dataset of free daily conversations.

This comparison showed that 19 out of 24 features showed a statistically consistent tendency with the results of the previous studies on connected speech data during neuropsychological tests (Table 3 and Multimedia Appendix 1). The other 5 features related to part-of-speech distribution (verb frequency, adjective ratio, and noun to verb ratio) and perseveration (average cosine distance and proportion of sentence pairs whose cosine distance is less than threshold 0.50) showed a statistically different tendency.

**Table 2:** Top 15 features of high discriminative power among linguistic features used in previous studies and repetition features. The table contains area under the receiver operating characteristic curve (AUC-ROC) score, effect size (Cohen d) with 95% CI, and *P* value of 2-sided t test with Bonferroni multiple testing correction.

Feature type	AUC-ROC^a^	Effect size (95% CI)	Adjusted *P* value
Topic repetition in two different conversations separated by *t* days interval (*T*=7)^b^	0.91	–1.76 (–2.15 to –1.36)	4.17E–17
Word repetition in two different conversations separated by *t* days interval (*T*=10)^b^	0.90	–1.67 (–2.06 to –1.29)	4.44E–17
Conjunction ratio	0.89	–2.03 (–2.33 to –1.73)	2.04E–41
Pronoun to noun ratio	0.87	–1.94 (–2.24 to –1.64)	3.17E–38
Topic repetition in two different conversations separated by *n* phone calls (*N*=9)^b^	0.86	–1.35 (–1.51 to –1.20)	2.75E–67
Noun ratio	0.86	1.38 (1.09 to 1.67)	4.28E–20
Word repetition in two different conversations separated by *n* phone calls (*N*=7)^b^	0.84	–1.22 (–1.36 to –1.08)	9.35E–66
Pronoun ratio	0.82	–1.50 (–1.79 to –1.21)	1.30E–23
Noun to verb ratio	0.81	0.63 (0.35 to 0.91)	2.73E–04
Honoré’s statistic	0.80	1.03 (0.75 to 1.32)	1.61E–11
Word repetition in single conversations^b^	0.79	–1.08 (–1.36 to –0.79)	1.57E–12
Topic repetition in single conversations^b^	0.78	–0.80 (–1.09 to –0.52)	7.62E–07
Conjunction frequency	0.77	–1.27 (–1.55 to –0.98)	4.22E–17
Noun frequency	0.75	0.71 (0.43 to 0.99)	1.66E–05
Adjective ratio	0.75	–1.06 (–1.34 to –0.77)	5.12E–12

^a^AUC-ROC: area under the receiver operating characteristic curve.

^b^Topic and word repetition features proposed by the authors.

**Table 3:** Comparison of the results of statistical analysis for the linguistic features between our study and previous studies. Our study analyzes speech data during daily conversations, while the previous studies analyzed connected speech data during neuropsychological tests. Sig and nonsig refer to significant and nonsignificant. For example, sig-nonsig in the inconsistent column indicates a feature that showed significant difference in the previous studies but not in our study. Cells contain the name of the corresponding features. Features whose statistical test results were not reported in the previous studies are excluded from this summary table. Information including *P* values of the statistical analysis in our study and the list of the previous studies is provided in Multimedia Appendix 1.

Feature category	Consistent (our study–previous studies)		Inconsistent (our study–previous study)	
	Sig-sig	Nonsig-nonsig	Sig-nonsig	Nonsig-sig
Part of speech (12)	Noun frequency Auxiliary verb frequency Noun ratio Verb ratio Pronoun ratio Auxiliary verb ratio Conjunction ratio Pronoun to noun ratio	Adverb ratio	Adjective ratio Noun to verb ratio	Verb frequency
Vocabulary richness (3)	Honoré’s statistic	Type-token ratio Brunét’s index	—	—
Syntactic complexity (7)	Mean length of sentence (utterance)	Total no of words No of sentences (utterances) No of characters Total dependency distance in a document Avg dependency distance per sentence Total no of distance in a document	—	—
Perseveration (2)	—	—	—	Avg cosine distance Cosine cutoff: 0.50

## Discussion

### Principal Findings

In response to the increasing demand for detecting dementia in everyday situations, we aimed to quantify language dysfunctions observed in AD from daily conversational data when the target is not performing neuropsychological or cognitive tasks. Although previous studies have succeeded in quantifying many aspects of language dysfunction, including grammatical and informational content as well as speech characteristics resulting from AD, they mainly investigated connected speech data collected while targets were engaged in neuropsychological and cognitive tasks; how language dysfunction in daily conversations can be quantified has not been sufficiently investigated. In this study, we focused on atypical repetition of words and topics reported by previous observational and descriptive studies as one of the prominent characteristics in everyday conversations of AD patients. Using conversational data of seniors with and without AD obtained from longitudinal follow-up in a regular monitoring service (from n=15 individuals including 2 AD patients at an average follow-up period of 16.1 months; 1032 conversational data items obtained during phone calls and approximately 221 person-hours), we investigated whether and how linguistic features related to word and topic repetition can be used for differentiating AD patients.

The results indicated that atypical repetition across different conversations could have a better discriminative power for AD compared to that within single conversations. We assume this atypical repetition across different conversations results from memory impairment that prevents speakers from remembering recent conversations. If so, the results suggest that the difference of memory performance between AD and healthy older adults might be larger after the lapse of some time than immediately, and linguistic features relating to atypical repetition could capture such difference even in daily conversations. In fact, deficits in episodic memory are one of the earliest detectable cognitive impairments in AD [53], and some experimental studies on episodic memory tasks such as word-list learning have reported that the forgetting rate passing from immediate to delayed recall in AD was significantly larger than that in age-matched controls [54]. These experimental studies seem to support our results. In addition, our results suggest that interval days between two conversations might be a more important parameter than the number of phone calls in terms of a discriminating power. These results might also be reasonable if the linguistic features relating to repetition can capture memory impairments resulting from pathological changes underlying AD. Furthermore, recent studies have reported accelerated long-term forgetting even in presymptomatic AD, which has garnered increased attention to detecting presymptomatic changes in AD [55]. From this perspective, our approach, which focuses on atypical repetition in daily conversations on different days, holds promise for detecting early signs of AD in everyday situations.

On the basis of comparisons with the standard linguistic features used in previous studies on connected speech data during neuropsychological tests, we found that features relating to atypical repetition in two conversations on different days had better AUC-ROC scores for differentiating AD than these linguistic features on speech data of daily conversations. The results suggest that repetition features across daily conversations on different days might be better indicators for detecting AD in everyday situations. We also compared the results of statistical analysis for each linguistic feature between previous studies on connected speech data during neuropsychological tests and our studies on daily conversations and found that about 80% of features (19 out of 24) showed a statistically consistent tendency. These results indicate that these linguistic features could be useful for inferring AD from speech data not only during neuropsychological tests but also during daily conversations. Features showing a statistically different tendency included those related to part-of-speech distributions and perseveration. In terms of the features of part-of-speech distribution, adjective ratio and noun to verb ratio showed significant difference between AD and controls in our study, while they were reported to have no significant difference in previous studies on connected speech data during neuropsychological tests [15,18,22,24]. In contrast, previous studies on speech data during semistructured interviews have reported that these two features were significant different [11,56]. Thus, language dysfunction in AD measured by these two features might be discriminative, especially in conversations in which the contents are less predetermined. As for the features related to perseveration, we found no significant difference in our dataset, while previous work reported the statistical significance of these features [22]. For example, one study used these features for measuring semantic similarity using a bag-of-words model in speech data during a picture description task and reported a significant difference between AD and controls [22,57]. Considering our results on the statistical analysis for these features, when we analyze unstructured and free conversational data such as daily conversations, language dysfunction in AD related to perseveration might need to be measured by combining a model for estimating the semantic topics (eg, BTM). In fact, the semantic similarity when using the BTM revealed a significant difference between AD and controls in our dataset.

In this study, we analyzed daily conversational data and showed that linguistic features, especially those related to repetition, can potentially be used for automatically detecting AD in a longitudinal and passive manner. Indeed, speech data collected in daily lives has gained increasing interest for clinical applications due to the improvement in audio quality recorded by portable devices and the expansion of voice-based interaction systems such as smartphones and smart speakers [4,58]. In particular, several studies that administrated cognitive and neuropsychological assessments over the phone have reported that speech features could be reliably extracted from phone recordings and used to build models for AD screening [59]. However, there are still relatively few studies on changes in speech features in daily conversations resulting from AD. Being capable of inferring AD from daily conversations would help with timely detection by frequent assessments with relatively short intervals, which might be difficult to do when using neuropsychological tests due to learning effects. From this perspective, we believe that the results of our study will help promote future efforts toward early detection of AD in everyday situations.

### Limitations

Our work has several limitations. First, the number of participants was small, especially for AD patients, although the follow-up periods and number of conversations for each participant were relatively long and large. The results of a post hoc power analysis revealed that we would need at least two additional AD patients to obtain more than 0.80 of the power. A second limitation is the lack of information about AD severity and stage. We analyzed conversational data collected from the users of a regular monitoring service, and as such, were not able to obtain information related to clinical assessments, such as Mini-Mental State Exam scores and biomarkers (aside from AD diagnosis provided by medical doctors). Third, the age and gender of the control and AD groups were not matched. These three limitations stem from the fact that our dataset was collected from real users of an actual monitoring service. To clarify the relevance of our results obtained from the datasets with these limitations, we comprehensively compared the statistical results of each of the 24 linguistic features between our study and more than 20 previous studies and confirmed that our dataset showed a statistically consistent tendency with the previous studies. In addition to this perspective, because our dataset represents invaluable data collected from a real service and consists of 1032 conversational data items at an average follow-up period of 16.1 months, we believe our results can provide useful information for future studies toward the early detection of AD in the real world. The fourth limitation is the use of transcribed conversation data after omitting information related to incomplete words and fillers. While previous studies on connected speech during neuropsychological tests reported that these features did not significantly differ between controls and AD patients [40], we feel that further investigation is required, especially on speech data in daily conversations. The fifth limitation was the specific type of conversational data used. In this study, we analyzed free conversation between older adults and communicators mainly about the lives of the older individuals, but we need further research to confirm our results and extend the scope into various types of daily conversation, such as phone call conversations with their friends and face-to-face family conversations.

### Conclusions

In summary, we investigated the daily conversational data of seniors with and without AD obtained from longitudinal follow-up in a regular monitoring service. We demonstrated that, while the linguistic features used in previous studies on connected speech data during neuropsychological tests can be used for detecting AD from daily conversations, the novel features related to repetition across conversations on different days could be a better indicator. We believe that our results can help to promote future efforts toward early detection of AD in everyday situations by taking advantage of speech data that can be collected in a passive, longitudinal manner.

## Appendix

Multimedia Appendix 1 Results of statistical analysis for the linguistic features in our dataset of daily conversations and the summary of the statistical analysis of these features in previous studies on speech data during neuropsychological tests.

## Abbreviations

AD: Alzheimer disease
AUC-ROC: area under the receiver operating characteristic curve
BI: Brunét’s index
BTM: biterm topic model
HS: Honoré’s statistic
TF-IDF: term frequency–inverse document frequency
TTR: type-token ratio

## References

[1] (2018). Alzheimer's Disease International.

[2] Prince M, Comas-Herrera A, Knapp M, Guerchet M, Karagiannidou M (2016). Alzheimer's Disease International.

[3] Teipel S, König A, Hoey J, Kaye J, Krüger F, Robillard JM, Kirste T, Babiloni C (2018). Use of nonintrusive sensor-based information and communication technology for real-world evidence for clinical trials in dementia. Alzheimers Dement.

[4] Kourtis LC, Regele OB, Wright JM, Jones GB (2019). Digital biomarkers for Alzheimer's disease: the mobile/ wearable devices opportunity. NPJ Digit Med.

[5] Kirshner HS (2012). Primary progressive aphasia and Alzheimer's disease: brief history, recent evidence. Curr Neurol Neurosci Rep.

[6] MacKay DG, James LE, Hadley CB (2008). Amnesic H.M.'s performance on the language competence test: parallel deficits in memory and sentence production. J Clin Exp Neuropsychol.

[7] van Velzen M, Garrard P (2013). From hindsight to insight—retrospective analysis of language written by a renowned Alzheimer's patient. Interdiscip Sci Rev.

[8] Oulhaj A, Wilcock GK, Smith AD, de Jager CA (2009). Predicting the time of conversion to MCI in the elderly: role of verbal expression and learning. Neurology.

[9] Ahmed S, de Jager CA, Haigh AF, Garrard P (2012). Logopenic aphasia in Alzheimer's disease: clinical variant or clinical feature?. J Neurol Neurosurg Psychiatry.

[10] Ahmed S, de Jager CA, Haigh A, Garrard P (2013). Semantic processing in connected speech at a uniformly early stage of autopsy-confirmed Alzheimer's disease. Neuropsychology.

[11] Bucks RS, Singh S, Cuerden JM, Wilcock GK (2000). Analysis of spontaneous, conversational speech in dementia of Alzheimer type: evaluation of an objective technique for analysing lexical performance. Aphasiology.

[12] Hoffmann I, Nemeth D, Dye CD, Pákáski M, Irinyi T, Kálmán J (2010). Temporal parameters of spontaneous speech in Alzheimer's disease. Int J Speech Lang Pathol.

[13] Guinn C, Habash A (2012). AAAI Fall Symposium: Artificial Intelligence for Gerontechnology.

[14] Ahmed S, Haigh AF, de Jager CA, Garrard P (2013). Connected speech as a marker of disease progression in autopsy-proven Alzheimer's disease. Brain.

[15] Beltrami D, Gagliardi G, Rossini Favretti R, Ghidoni E, Tamburini F, Calzà L (2018). Speech analysis by natural language processing techniques: a possible tool for very early detection of cognitive decline?. Front Aging Neurosci.

[16] Bschor T, Kühl KP, Reischies FM (2001). Spontaneous speech of patients with dementia of the Alzheimer type and mild cognitive impairment. Int Psychogeriatr.

[17] Carlomagno S, Santoro A, Menditti A, Pandolfi M, Marini A (2005). Referential communication in Alzheimer's type dementia. Cortex.

[18] Croisile B, Ska B, Brabant MJ, Duchene A, Lepage Y, Aimard G, Trillet M (1996). Comparative study of oral and written picture description in patients with Alzheimer's disease. Brain Lang.

[19] Cuetos F, Arango-Lasprilla JC, Uribe C, Valencia C, Lopera F (2007). Linguistic changes in verbal expression: a preclinical marker of Alzheimer's disease. J Int Neuropsychol Soc.

[20] Feyereisen P, Berrewaerts J, Hupet M (2007). Pragmatic skills in the early stages of Alzheimer's disease: an analysis by means of a referential communication task. Int J Lang Commun Disord.

[21] Forbes-McKay K, Shanks MF, Venneri A (2013). Profiling spontaneous speech decline in Alzheimer's disease: a longitudinal study. Acta Neuropsychiatr.

[22] Fraser KC, Meltzer JA, Rudzicz F (2016). Linguistic features identify Alzheimer's disease in narrative speech. J Alzheimers Dis.

[23] Hernández-Domínguez L, Ratté S, Sierra-Martínez G, Roche-Bergua A (2018). Computer-based evaluation of Alzheimer's disease and mild cognitive impairment patients during a picture description task. Alzheimers Dement (Amst).

[24] Kavé G, Levy Y (2003). Morphology in picture descriptions provided by persons with Alzheimer's disease. J Speech Lang Hear Res.

[25] Kavé G, Goral M (2016). Word retrieval in picture descriptions produced by individuals with Alzheimer's disease. J Clin Exp Neuropsychol.

[26] March EG, Wales R, Pattison P (2006). The uses of nouns and deixis in discourse production in Alzheimer's disease. J Neurolinguistics.

[27] Mueller KD, Koscik RL, Hermann BP, Johnson SC, Turkstra LS (2018). Declines in connected language are associated with very early mild cognitive impairment: results from the Wisconsin Registry for Alzheimer's Prevention. Front Aging Neurosci.

[28] Nicholas M, Obler LK, Albert ML, Helm-Estabrooks N (1985). Empty speech in Alzheimer's disease and fluent aphasia. J Speech Hear Res.

[29] Orimaye S, Wong J, Golden K (2014). Learning predictive linguistic features for Alzheimer's disease and related dementias using verbal utterances.

[30] Orimaye SO, Wong JS, Golden KJ, Wong CP, Soyiri IN (2017). Predicting probable Alzheimer's disease using linguistic deficits and biomarkers. BMC Bioinformatics.

[31] Roark B, Mitchell M, Hosom J, Hollingshead K, Kaye J (2011). Spoken language derived measures for detecting mild cognitive impairment. IEEE Trans Audio Speech Lang Process.

[32] Sajjadi SA, Patterson K, Nestor PJ (2014). Logopenic, mixed, or Alzheimer-related aphasia?. Neurology.

[33] Shimada M, Meguro K, Yamazaki H, Horikawa A, Hayasaka C, Yamaguchi S, Yamaguchi K, Katsuyama N, Nakano M, Yamadori A (1998). Impaired verbal description ability assessed by the Picture Description Task in Alzheimer's disease. Arch Gerontol Geriatr.

[34] Yancheva M, Fraser K, Rudzicz F (2015). Using linguistic features longitudinally to predict clinical scores for Alzheimer's disease and related dementias.

[35] Henry JD, Crawford JR, Phillips LH (2004). Verbal fluency performance in dementia of the Alzheimer's type: a meta-analysis. Neuropsychologia.

[36] Kavé G, Goral M (2018). Word retrieval in connected speech in Alzheimer's disease: a review with meta-analyses. Aphasiology.

[37] König A, Satt A, Sorin A, Hoory R, Toledo-Ronen O, Derreumaux A, Manera V, Verhey F, Aalten P, Robert PH, David R (2015). Automatic speech analysis for the assessment of patients with predementia and Alzheimer's disease. Alzheimers Dement (Amst).

[38] Lunsford R, Heeman P (2015). Using linguistic indicators of difficulty to identify mild cognitive impairment.

[39] Chinaei H, Currie LC, Danks A, Lin H, Mehta T, Rudzicz F (2017). Identifying and avoiding confusion in dialogue with people with Alzheimer's disease. Computational Linguistics.

[40] Boschi V, Catricalà E, Consonni M, Chesi C, Moro A, Cappa SF (2017). Connected speech in neurodegenerative language disorders: a review. Front Psychol.

[41] Cook C, Fay S, Rockwood K (2009). Verbal repetition in people with mild-to-moderate Alzheimer Disease: a descriptive analysis from the VISTA clinical trial. Alzheimer Dis Assoc Disord.

[42] Kudo T (2005). Mecab: yet another part-of-speech and morphological analyzer.

[43] Honoré A (1979). Some simple measures of richness of vocabulary.

[44] Yan X, Guo J, Lan Y, Cheng X (2014). BTM: topic modeling over short texts. IEEE Transact Knowl Data Engineer.

[45] Blei D, Ng A, Jordan M (2003). Latent dirichlet allocation. J Mach Learn Res.

[46] Goodglass H, Kaplan E (1983). The Boston Diagnostic Aphasia Examination.

[47] Swinburn K, Porter G, Howard D (2004). Comprehensive Aphasia Test.

[48] Wechsler D (1997). Wechsler Memory Scale. Third Edition Manual.

[49] Kudo T Cabocha: yet another Japanese dependency structure analyzer.

[50] Liu H, Xu C, Liang J (2017). Dependency distance: a new perspective on syntactic patterns in natural languages. Phys Life Rev.

[51] Masrani V, Murray G, Field T, Carenini G (2017). Detecting dementia through retrospective analysis of routine blog posts by bloggers with dementia. BioNLP.

[52] Nakagawa S, Cuthill IC (2007). Effect size, confidence interval and statistical significance: a practical guide for biologists. Biol Rev Camb Philos Soc.

[53] Greene JD, Baddeley AD, Hodges JR (1996). Analysis of the episodic memory deficit in early Alzheimer's disease: evidence from the doors and people test. Neuropsychologia.

[54] Carlesimo GA, Mauri M, Graceffa AM, Fadda L, Loasses A, Lorusso S, Caltagirone C (1998). Memory performances in young, elderly, and very old healthy individuals versus patients with Alzheimer's disease: evidence for discontinuity between normal and pathological aging. J Clin Exp Neuropsychol.

[55] Weston PSJ, Nicholas JM, Henley SMD, Liang Y, Macpherson K, Donnachie E, Schott JM, Rossor MN, Crutch SJ, Butler CR, Zeman AZ, Fox NC (2018). Accelerated long-term forgetting in presymptomatic autosomal dominant Alzheimer's disease: a cross-sectional study. Lancet Neurol.

[56] Jarrold W, Peintner B, Wilkins D (2014). Aided diagnosis of dementia type through computer-based analysis of spontaneous speech.

[57] Fraser K (2016). Automatic Text and Speech Processing for the Detection of Dementia [Thesis].

[58] Manfredi C, Lebacq J, Cantarella G, Schoentgen J, Orlandi S, Bandini A, DeJonckere PH (2017). Smartphones Offer New Opportunities in Clinical Voice Research. J Voice.

[59] Van Mierlo LD, Wouters H, Sikkes SA, Van der Flier WM, Prins ND, Bremer JA, Koene T, Van Hout HPJ (2017). Screening for mild cognitive impairment and dementia with automated, anonymous online and telephone cognitive self-tests. J Alzheimers Dis.

