# Supplementary material for: Atypical Repetition in Daily Conversation on Different Days for Detecting Alzheimer Disease: Evaluation of Phone-Call Data From a Regular Monitoring Service
Source: JMIR Ment Health. 2020 Jan 14;7(1):e16790. doi: 10.2196/16790 (PMC6996758; doi:10.2196/16790)
Supplement: Multimedia Appendix 1 [file mental_v7i1e16790_app1.docx]

**Multimedia Appendix** **1.** Results of statistical analysis for the linguistic features in our dataset of daily conversations and the summary of the statistical analysis of these features in previous studies on speech data during neuropsychological tests. The results of our study include area under the receiver operating characteristic curve (AUC-ROC), effect size (Cohen *d*) with 95% CI, and *P* value of two-sided t-test with Bonferroni multiple testing correction. An asterisk (*) in the adjusted *P* values indicates significant difference, which is below .01. The summary of the results in previous studies includes the ratio between the studies reporting significant between-group differences and the number of studies reporting the results of the statistical analysis for each feature. A dagger (†) indicates the specific studies that reported significant between-group differences. A double dagger (‡) indicates more than half of the studies considered significant features. Citation number assigned in the reference is the same as the body of this paper.

| **Features** | **Our Study (Dataset of daily conversations)** | | | **Previous Study (Dataset of speech data during neuropsychological tests)** | |
| --- | --- | --- | --- | --- | --- |
|  | **AUC-ROC score** | **Effect size [95% CI]** | **Adjusted**  ***P* value** | **Ratio** | **References** |
| **Part-of-speech** | | | | | |
| Noun frequency | 0.75 | 0.71 [ 0.43, 0.99] | 1.66E-05* | 1/1‡ | [23] Hernández-Domínguez et al., 2018^e^† |
| Verb frequency | 0.57 | 0.24 [–0.04, 0.51] | 1 | 1/1‡ | [22] Fraser et al., 2016^d^† |
| Adjective frequency | 0.58 | –0.29 [–0.57, –0.01] | 1 | unknown | [23] Hernández-Domínguez et al., 2018^c,e^ |
| Auxiliary verb  frequency | 0.74 | 0.61 [0.34, 0.89] | 5.07E-04* | 1/1‡ | [23] Hernández-Domínguez et al., 2018 ^e^† |
| Conjunction  frequency | 0.77 | –1.27 [–1.55, –0.98] | 4.22E-17* | unknown | [23] Hernández-Domínguez et al., 2018^c,e^ |
| Noun ratio | 0.86 | 1.38 [ 1.09, 1.67] | 4.28E-20* | 3/6‡ | [18] Croisile et al., 1996  [26] March et al., 2006†  [14] Ahmed et al., 2013^e^  [22] Fraser et al., 2016^d^†  [25] Kavè and Goral, 2016†  [15] Beltrami et al., 2018^e^ |
| Verb ratio | 0.69 | –0.62 [–0.9, –0.34] | 3.92E-04* | 2/4‡ | [18] Croisile et al., 1996  [14] Ahmed et al., 2013^e^  [34] Yancheva et al., 2015†  [22] Fraser et al., 2016^d^† |
| Adjective ratio | 0.75 | –1.06 [–1.34, –0.77] | 5.12E-12* | 0/3 | [18] Croisile et al., 1996  [22] Fraser et al., 2016^d^  [15] Beltrami et al., 2018^e^ |
| Pronoun ratio | 0.82 | –1.50 [–1.79, –1.21] | 1.30E-23* | 4/5‡ | [14] Ahmed et al., 2013^e^†  [34] Yancheva et al., 2015†  [22] Fraser et al., 2016^d^  [25] Kavè and Goral, 2016†  [15] Beltrami et al., 2018^e^† |
| Adverb ratio | 0.61 | –0.37 [–0.65, –0.09] | 3.19E-01 | 0/3 | [18] Croisile et al., 1996  [22] Fraser et al., 2016^d^  [15] Beltrami et al., 2018^e^ |
| Auxiliary verb ratio | 0.71 | 0.69 [ 0.41, 0.97] | 3.92E-05* | 1/2‡ | [19] Cuetos et al., 2007^e^  [22] Fraser et al., 2016^d^† |
| Conjunction ratio | 0.89 | –2.03 [–2.33, –1.73] | 2.04E-41* | 2/4‡ | [28] Nicholas et al., 1985†  [22] Fraser et al., 2016^d^  [15] Beltrami et al., 2018^e^  [23] Hernández-Domínguez et al., 2018†^e^ |
| Noun-to-verb ratio | 0.81 | 0.63 [ 0.35, 0.91] | 2.73E-04* | 0/2 | [24] Kavè and Levy, 2003  [22] Fraser et al., 2016^d^ |
| Pronoun-to-noun ratio | 0.87 | –1.94 [–2.24, –1.64] | 3.17E-38* | 2/2‡ | [24] Kavè and Levy, 2003†  [22] Fraser et al., 2016^d^† |
| **Vocabulary Richness** | | | | | |
| Type-token ratio | 0.51 | –0.02 [–0.29, 0.26] | 1 | 0/5 | [29] Orimaye et al., 2014  [34] Yancheva et al., 2015  [22] Fraser et al., 2016^d^  [25] Kavè and Goral, 2016  [15] Beltrami et al., 2018^e^ |
| Brunét’s index | 0.50 | 0.00 [–0.27, 0.28] | 1 | 0/2 | [22] Fraser et al., 2016^d^  [15] Beltrami et al., 2018^e^ |
| Honoré’s statistic | 0.79 | 1.03 [ 0.75, 1.32] | 5.73E-11* | 4/4‡ | [34] Yancheva et al., 2015†  [22] Fraser et al., 2016^d^†  [15] Beltrami et al., 2018^e^†  [23] Hernández-Domínguez et al., 2018 ^e^† |

| **Syntactic Complexity** | | | | | |
| --- | --- | --- | --- | --- | --- |
| Total no. of words | 0.65 | 0.44 [ 0.17, 0.72] | 5.86E-02 | 1/14 | [28] Nicholas et al., 1985  [18] Croisile et al., 1996†  [33] Shimada et al., 1998  [16] Bschor et al., 2001  [17] Carlomagno et al., 2003  [20] Feyereisen et al., 2007  [31] Roark et al., 2011^e^  [32] Sajjadi et al., 2012^e^  [10] Ahmed et al., 2013^e^  [29] Orimaye et al., 2014  [34] Yancheva et al., 2015  [22] Fraser et al., 2016^d^  [30] Orimaye et al., 2017  [27] Mueller et al., 2018^e^ |
| Mean length of sentence (utterance) (MLS or MLU) ^a^ | 0.67 | 0.53 [ 0.25, 0.81] | 6.68E-03* | 5/10‡ | [19] Cuetos et al., 2007^e^  [31] Roark et al., 2011^e^  [14] Ahmed et al., 2013^e^  [21] Forbes-Mckay et al., 2013  [29] Orimaye et al., 2014†  [34] Yancheva et al., 2015†  [22] Fraser et al., 2016^d^  [30] Orimaye et al., 2017†  [27] Mueller et al., 2018^e^†  [15] Beltrami et al., 2018^e^† |
| Total no. of sentences (utterances) in a document ^a^ | 0.55 | 0.25 [–0.02, 0.53] | 1 | 1/4 | [19] Cuetos et al., 2007^e^  [31] Roark et al., 2011^e^  [29] Orimaye et al., 2014†  [30] Orimaye et al., 2017 |
| Total no. of characters in a document | 0.67 | 0.50 [ 0.23, 0.78] | 1.27E-02 | 0/1 | [30] Orimaye et al., 2017 |
| Avg. dependencies per sentence | 0.58 | 0.25 [–0.02, 0.53] | 1 | 0/1 | [30] Orimaye et al., 2017 |
| Total no. of dependencies in a document | 0.61 | 0.37 [ 0.09, 0.65] | 3.23E-01 | 0/1 | [30] Orimaye et al., 2017 |
| Total dependency distance in a document | 0.61 | 0.36 [ 0.08, 0.64] | 3.81E-01 | 0/1 | [30] Orimaye et al., 2017 |

| **Perseveration** | | | | | |
| --- | --- | --- | --- | --- | --- |
| Average cosine distance | 0.61 | -0.28 [–0.55, 0.00] | 1 | 1/1‡ | [22] Fraser et al., 2016^d^† |
| Minimum cosine distance | 0.54 | 0.40 [ 0.13, 0.68] | 1.51E-01 | unknown | [22] Fraser et al., 2016^d^ |
| Cosine cutoff: 0 ^b^ | 0.51 | –0.09 [–0.36, 0.19] | 1 | unknown | [22] Fraser et al., 2016^d^ |
| Cosine cutoff: 0.3 ^b^ | 0.70 | 0.45 [ 0.17, 0.73] | 5.08E-02 | unknown | [22] Fraser et al., 2016^d^ |
| Cosine cutoff: 0.5 ^b^ | 0.60 | 0.19 [–0.09, 0.47] | 1 | 1/1‡ | [22] Fraser et al., 2016^d^† |

a. Utterance is identified to start from the beginning of verbal communication to the next verbal pause length. We defined utterance as the unit segmented by punctuation marks (period, question mark, and exclamation mark), as we did not have audio data to extract the verbal pause length.

b. Cosine cutoff means the number of utterance pairs with cosine distance equal to zero, less than 0.3, and less than 0.5 (normalized by the total number of pairs) [22].

c. The paper reported the correlations of features with the cognitive impairment diagnosis. It reported the 17 strongest features, but the results of the other features were not mentioned. For the features without a statistical result, we marked significance as “unknown”.

d. The result of this paper is the combination of the review article [40] and our survey. The paper reported the top 50 features with strong correlations with the factors along with the correlations with diagnosis. In addition to the result of the review [40], we added the unlisted features by defining the features included in these top 50 as significant, and “unknown” for the rest.

e. Paper includes early stage of AD (e.g., mild cognitive impairments) in the result of statistical analysis.
